# Crosstalk from Non-Cancerous Mitochondria Can Inhibit Tumor Properties of Metastatic Cells by Suppressing Oncogenic Pathways

**DOI:** 10.1371/journal.pone.0061747

**Published:** 2013-05-09

**Authors:** Benny Abraham Kaipparettu, Yewei Ma, Jun Hyoung Park, Tin-Lap Lee, Yiqun Zhang, Patricia Yotnda, Chad J. Creighton, Wai-Yee Chan, Lee-Jun C. Wong

**Affiliations:** 1 Department of Molecular and Human Genetics, Baylor College of Medicine, Houston, Texas, United States of America; 2 Dan L. Duncan Cancer Center, Baylor College of Medicine, Houston, Texas, United States of America; 3 Section on Developmental Genomics, Eunice Kennedy Shriver National Institute of Child Health and Human Development, National Institutes of Health, Bethesda, Maryland, United States of America; 4 School of Biomedical Sciences, Faculty of Medicine, The Chinese University of Hong Kong, Hong Kong, China; 5 Center for Gene Therapy, Baylor College of Medicine, Houston, Texas, United States of America; Roswell Park Cancer Institute, United States of America

## Abstract

Mitochondrial-nucleus cross talks and mitochondrial retrograde regulation can play a significant role in cellular properties. Transmitochondrial cybrid systems (cybrids) are an excellent tool to study specific effects of altered mitochondria under a defined nuclear background. The majority of the studies using the cybrid model focused on the significance of specific mitochondrial DNA variations in mitochondrial function or tumor properties. However, most of these variants are benign polymorphisms without known functional significance. From an objective of rectifying mitochondrial defects in cancer cells and to establish mitochondria as a potential anticancer drug target, understanding the role of functional mitochondria in reversing oncogenic properties under a cancer nuclear background is very important. Here we analyzed the potential reversal of oncogenic properties of a highly metastatic cell line with the introduction of non-cancerous mitochondria. Cybrids were established by fusing the mitochondria DNA depleted 143B TK- ρ0 cells from an aggressive osteosarcoma cell line with mitochondria from benign breast epithelial cell line MCF10A, moderately metastatic breast cancer cell line MDA-MB-468 and 143B cells. In spite of the uniform cancerous nuclear background, as observed with the mitochondria donor cells, cybrids with benign mitochondria showed high mitochondrial functional properties including increased ATP synthesis, oxygen consumption and respiratory chain activities compared to cybrids with cancerous mitochondria. Interestingly, benign mitochondria could reverse different oncogenic characteristics of 143B TK^-^ cell including cell proliferation, viability under hypoxic condition, anti-apoptotic properties, resistance to anti-cancer drug, invasion, and colony formation in soft agar, and *in vivo* tumor growth in nude mice. Microarray analysis suggested that several oncogenic pathways observed in cybrids with cancer mitochondria are inhibited in cybrids with non-cancerous mitochondria. These results suggest the critical oncogenic regulation by mitochondrial-nuclear cross talk and highlights rectifying mitochondrial functional properties as a promising target in cancer therapy.

## Introduction

Cancer cells adapt to hypoxic conditions during progressive tumor cell growth by shifting the burden of energy metabolism from oxidative phosphorylation to glycolysis, referred to as the Warburg effect [1]. The regulation of nuclear gene expression by the mitochondrial genome, through ‘mitochondria retrograde signaling’, allows the organelles to coordinate their function with the nucleus. Tumor cells continue to utilize glycolysis as the major energy source even in culture under normoxic conditions [2], suggesting that possible stable genetic or epigenetic changes have occurred in cancer cells. In addition, cancer mitochondria without detectable genetic changes may transmit oncogenic signals to the nucleus and initiate mitochondrial retrograde regulation leading to the bidirectional communication between the two genomes [3].

In order to investigate the specific mitochondrial contribution to tumor properties, the effect of nuclear genes must be excluded. Transmitochondrial cybrid system is an excellent approach to achieve this goal [4]–[9]. Several studies used this exciting technology mostly to show the functional and pathogenic significance of specific mitochondrial DNA (mtDNA) mutations or variants [5], [10]. The mtDNA is known to mutate frequently in a variety of cancers but most of these mtDNA alterations, except a few, are without any known functional relevance and may simply reflect the genomic instability of tumor cells. Even without the presence of known deleterious mtDNA mutations, studies have shown that metastatic mitochondria can enhance the tumor property of a cancer cell and make them metastatic [9], [11]. However, from a therapeutic point of view, in order to target diseased mitochondria, it is important to know whether non-cancerous functional mitochondria can reverse the oncogenic property of metastatic cells. If so, targeting diseased mitochondria or rectifying the functional defect of normal mitochondria may provide a critical druggable area for cancer therapy. In this study, we have asked an interesting question whether non-cancerous mitochondria can reverse the oncogenic properties of an aggressive cancer cell. Under a defined cancerous nuclear background, we compared mitochondria from non-cancerous, moderately metastatic breast cells in a highly metastatic nuclear background with mitochondria from highly aggressive cancer cell as control. Even under the same nuclear background, mitochondria from non-cancerous cells could inhibit several oncogenic pathways, reverse the oncogenic properties and enhance therapeutic response of the cancer cells. This highlights the significance of mitochondria as a critical regulator of cellular cancer property and a potential target for anticancer therapy.

## Materials and Methods

### Ethics Statement on Animal Experiments

All animal procedures were approved by Institutional Animal Care and Use Committee at Baylor College of Medicine and performed in accordance with NIH guidelines for the ethical treatment of animals.

### Cybrids

Immortalized non-cancerous mammary epithelial MCF10A cells, breast cancer MDA-MB-468 cells and metastatic osteosarcoma-derived 143B TK^-^ cells (143B) were obtained from American Type Culture Collection (Manassas, VA). A detailed protocol for the generation of *ρ^0^* cells and cybrids has recently published [3], [6], [8]. Briefly, mtDNA depleted 143B ρ^0^ cells were used as the nuclear donor. For generating cybrids, mitochondrial donor cells (MCF10A, MDA-MB-468 and 143B TK-) were enucleated by overnight actinomycin-D treatment and fused with the 143B ρ^0^ cells using 45% polyethylene glycol (MW 1450; Sigma cat P-5402) for 60 seconds. One day following fusion, cells were cultured in selection medium with bromodeoxyuridine (BrdU) but without uridine [3], [12]. Unfused control 143B ρ^0^ cells could not survive without uridine and mitochondria donor cells were killed by BrdU. Cybrids containing mitochondria derived from MCF10A, MDA-MB-468 and 143B cells with 143B ρ^0^ cells as nuclear background are named as 10A/143B, 468/143B and 143B/143B cybrids, respectively.

### Quantification and Sequence Analysis of mtDNA and Validation of Nuclear DNA

Total genomic DNA was extracted by phenol/chloroform method and the 16.6-kb mitochondrial genome was amplified by 24 pairs of overlapping primers as published before [13]–[15]. qPCR was used to quantify mtDNA copy number for verifying the *ρ^0^* condition and to quantify mtDNA content in the cybrids according to published procedures [16], [17]. Only cybrid clones containing comparable mtDNA copy number were used for experiments. Whole genome mtDNA and reference nuclear DNA sequence analysis was performed using BigDye terminator (version 3.1) cycle sequencing reagent kit on an ABI 3730XL DNA Analyzer (Foster City, CA). The results of DNA sequences were compared with published Cambridge reference sequence from http://www.mitomap.org. Nuclear source of cybrids was confirmed by genotyping.

### Reactive Oxygen Species (ROS) Production

The cells were seeded and grown in glucose or galactose medium for 24 hours in 24-well plates (2×10^4^ per well). ROS production was measured 30 minutes after loading with 5 µmol/L 2′,7′-dichlorodihydrofluorescein diacetate (DCFH-DA) [18]. After washing with PBS buffer, cells were trypsinized and suspended in the PBS buffer. Fluorescence measurements at excitation and emission wavelengths of 485 and 535 nm, respectively, were carried out with a Tecan’s Infinite M200® microplate reader (Mannedorf, Switzerland).

### ATP Synthesis

The ATP synthesis was measured by the luciferin/luciferase assay [3]. Cells were incubated with 5 mmol/L malate plus 5 mmol/L glutamate (complex I–driven substrates), or with 10 mmol/L succinate (complex II–driven substrate) plus 2 µg/mL rotenone (complex I inhibitor), and 0.2 mmol/L ADP for 3 minutes in the presence or absence of 10 µg/ml oligomycin. The rate of oligomycin sensitive ATP synthesis was analyzed using Tecan’s Infinite M200® and expressed as nmol ATP produced/min/mg protein.

### Western Blot Analysis

Proteins were separated by SDS-PAGE on a 12% gel. Specific antibodies used for Western Blot analysis and chemiluminescence were detected using horseradish peroxidase-labeled as secondary antibody (Bio-Rad, CA). Mitochondrial proteins: ATP synthase subunit alpha (F1α), Core 2, FeS subunit (Ip), COXII, ND6, and Porin (all from Mitoscience, OR). Apoptotic proteins: cleaved caspase 3 and poly (ADP-ribose) polymerase (PARP) (Cell Signaling, MA). β-actin and Porin were used as loading controls for nuclear and mitochondrial encoded proteins respectively [3].

### Cell Proliferation Assay

Cell proliferation was measured at normoxia (21% O_2_) or hypoxia condition (1% O_2_) using colorimetric assay based on the measurement of BrdU incorporation (4 hours) during DNA synthesis, according to the manufacturer’s protocol (Roche Applied Science, Mannheim, Germany).

### TUNEL Assay

Apoptosis was analyzed by TUNEL assay using the DeadEnd™ Fluorometric TUNEL system (Promega, WI, USA) according to manufacturer’s instructions [19], [20].

### Anticancer Drug Treatment and MTT Assay

After overnight culturing of 5×10^3^ cybrids in a 96well plate, the cybrids were treated with vehicle, doxorubicin (0.1 µM) under normoxic (21% O_2_) and hypoxic (1% O_2_) conditions for another 24 h. Cell viability was analyzed by MTT assay using Tecan Infinite M200 according to published protocol [21], [22].

### Colony Formation in Soft Agar

Six-well plate with 0.5% agar in DMEM medium as the bottom layer was used for soft agar colony formation assay. For each well, 5×10^3^ cybrids suspended in DMEM medium with 0.35% agarose were plated as top layer and incubated at 37°C for 3–4 weeks. Colonies were stained with 0.005% Crystal violet and counted. The number of colonies for each cybrid was plotted as the mean ± SD.

### Matrigel™ Invasion Assay

The invasive growth capability of cybrids was quantified using BD BioCoat™ Matrigel™ Invasion Chamber (6-well) (Becton Dickinson Biosciences, MA, USA) according to the manufacturer’s protocol [23]. After trypsinization, 5×10^4^ cells in 0.5 ml medium were added to each well in triplicate. After 24 hours, the cells invaded to the lower chamber were fixed and stained with 100% methanol and 1% Toluidine blue. The number of invaded cells was counted using a microscope and the percentages of Matrigel matrix-invading cells were calculated compared to the uncoated control membrane.

### Microarray Gene Expression Analysis and Validation by qPCR

Gene expression profile of 10A/143B and 468/143B cybrids were compared using Affymetrix human U133 Plus 2.0 arrays in triplicate according to manufacturer’s instructions. Briefly, RNA from 10A/143B and 468/143B cybrids were extracted in triplicate and 5 µg of total RNA was used for first- and second-strand cDNA synthesis. The microarray hybridization was performed in Affymetrix U133 Plus 2.0 microarray chips at Affymetrix F450 fluidics station using the EukGE-WS2v5_450 protocol and scanned with an Affymetrix GeneChip 7 G scanner (Affymetrix, Santa Clara, CA). The data was analyzed by the statistical core facility at Dan L. Duncan Cancer Center at Baylor College of Medicine. Genes were defined significantly altered between the two cybrids with t-test P<0.01, fold change>2 or <0.5; global differences far exceeded chance expected. The gene expression data set was further subjected to pathway signature analysis to find putative differential pathway activation events. Pathway-associated gene signatures were defined, using public data: the p53 signature consisted of canonical bound and up-regulated p53 gene targets, as catalogued in the p53 IARC database (http://www-p53.iarc.fr/TargetGenes.html), and the other gene signatures examined were previously catalogued and described [24]. Using the set of unique genes represented in the dataset (where multiple probes referred to the same gene, the probe with the highest variability was used to represent the gene), and with expression values centered on the mean across samples, the average expression for the genes “up” in the signature was subtracted from the average for the genes “down,” in order to compute a summary pathway score for a given signature and sample profile. For randomly selected genes, mRNA expression differences observed in the microarray analysis were further validated by q-RT-PCR using primers described in Table S1.

### *In vivo* Tumor Growth Analysis

Tumor growth was assayed for cybrids were performed using nu/nu female nude mice (Charles River Laboratories, MA, USA). Four mice were used for each cybrid group. About 1.5 million cells each of the same cybrids were mixed with Matrigel (300 ul cell suspension in PBS with 300 ul reduced growth factor Matrigel), and injected into both the left and right flanks of the animals using a 26 gauge needle. Tumor size was measured twice a week with caliper and tumor volume was calculated using the formula LW^2^/2 (L and W representing the length and width of tumors) [25]. The experiment was completed when the mean of tumor volume in any of the control mice (143B/143B) exceeded 1.0 cm^3^. At the end of the experiment, mice were euthanized by exposure to CO_2_ and primary tumors were excised and weighed.

### Statistics

The results were presented as the mean±SD or SEM. Statistical analysis was performed using *t* test and ANOVA. P-value <0.05 was considered as significant.

## Results and Discussion

The cybrid technology described here allows the investigation of the effect mitochondria derived from cancer or non-cancer cells on mitochondrial function and oncogenic properties in a common nuclear background. Not all cells can survive under mitochondria depleted conditions. Here we used 143B TK^-^ cells as nuclear donor, which is one of the most well characterized cell line for cybrid studies and the first established human ρ0 cell line.

### Non-cancerous Cells and their Cybrids Exhibit Better Mitochondrial Functional Properties Compared to Cancer Mitochondria

**Figure 1:**
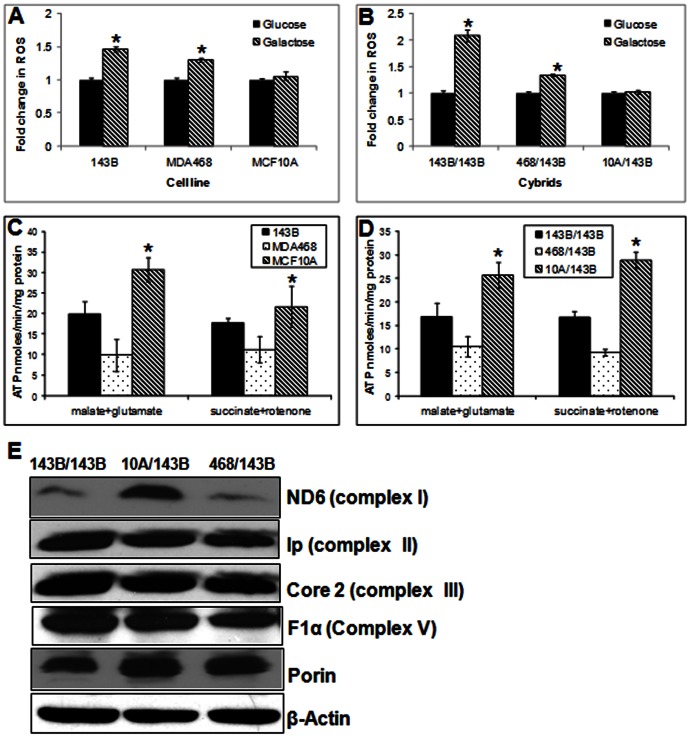
ROS production and ATP synthesis. (A) ROS production in parental cells. After Cells were grown in DMEM-glucose or DMEM-galactose medium for 24 h and ROS were measured using DCFH-DA fluorescent dye. Data expressed as DCF fluorescence/mg protein. (B) ROS production in cybrid cells. (C) Mitochondrial ATP synthesis rate in parental cells. ATP synthesis was driven by complex I substrates (glutamate plus malate) or complex II substrate (succinate) in the presence of complex I inhibitor (rotenone). The rates of ATP synthesis are expressed as nmoles ATP/min/mg protein. (D) Mitochondrial ATP synthesis rate in cybrids. (E) Western blot analysis of representative protein subunits of the mitochondria respiratory complexes in cybrids. β-actin and porin were used as loading controls for nuclear and mitochondria proteins respectively. (* = P<0.05).

We first analyzed the mitochondrial function of parental mitochondrial donor cell lines and their cybrids. Since the leakage of ROS is an indication of defective mitochondrial function, ROS production was analyzed in all parental cell lines using cell-permeable probe, DCFH-DA. Under metabolic stress, such as in the absence of glucose, cells are forced to solely depend on mitochondrial oxidative phosphorylation for energy supply [22]. Therefore, if there is defect in mitochondrial respiration, cells are expected to produce increased amount of ROS in the galactose medium [25]. As expected both cancer cell lines (143B and MDA-MB-468) produced significantly higher (p<0.05) ROS in galactose medium compared to glucose medium. However, there was no significant increase in ROS production for MCF10A cells in the galactose medium (Fig. 1A). We then analyzed the cybrids and obtained a similar response as observed with the parental cells. For cybrids with mitochondria from benign MCF10A cells, there was no significant increase in ROS level in galactose medium. However, cybrids with cancer mitochondria showed significantly increased ROS level in galactose medium (Fig. 1B). To further investigate the site of failure in the respiratory chain responsible for the impaired oxidative phosphorylation, the ATP synthesis rates were measured in the presence of complex I substrates (glutamate plus malate), or complex II substrate (succinate). As shown in Fig. 1C, ATP synthesis rates were significantly higher in benign cells compared to the cancer cells in the presence of complex I (p<0.01) or complex II substrates (p<0.01). As expected, cybrid cells also showed similar results for ATP synthesis as observed with the parental cells. In the presence of either complex I substrates or complex II substrates, ATP synthesis rate was markedly increased in 10A/143B cybrids compared to 143B/143B and 468/143B cybrids (p<0.01) (Fig. 1D). These results suggest that cancer mitochondria probably have altered function affecting multiple complexes in the respiratory chain. Western blot analysis showed that compared to cybrids with cancer mitochondria, the mtDNA-encoded mitochondrial complex I ND6 subunit of mitochondrial NADH dehydrogenase protein was significantly increased in MCF10A/143 cybrids but no difference in nuclear encoded proteins like F1α, Core2 and Ip (Fig. 1E). Thus, introduction of mitochondria from non-cancerous MCF10A could at least partially increase the expression of mtDNA encoded respiratory chain proteins, thus, rectify the reduced mitochondrial function in 143B cancer cell.

### Non-cancerous Mitochondria can Reverse the Oncogenic Properties of Metastatic Cell

To understand the functional significance of mitochondrial retrograde regulation from non-cancerous mitochondria on the tumorigenic properties of a metastatic nucleus, we compared the cybrids for different oncogenic properties.

### Cell Proliferation and Viability under Hypoxic Condition

**Figure 2:**
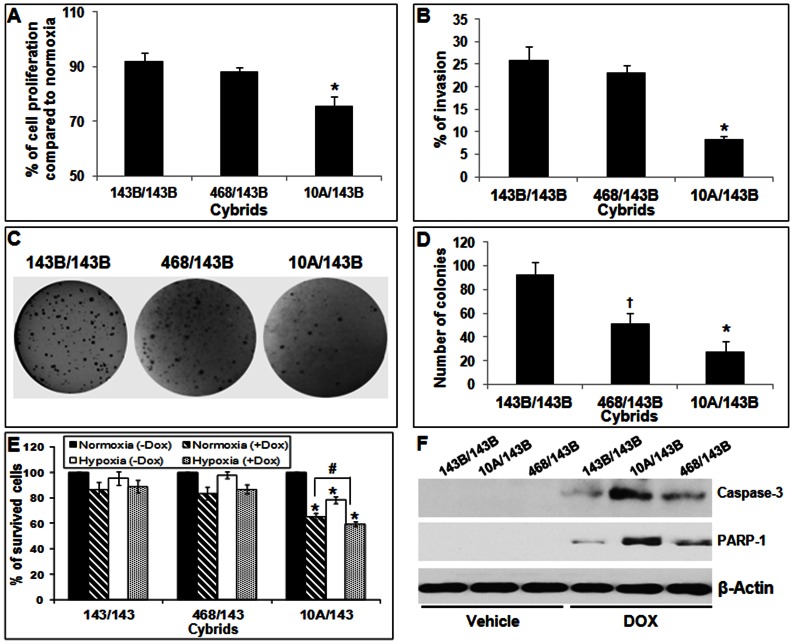
Comparison of *in vitro* oncogenic properties in cybrids. (A) Percentage of cell proliferation in cybrids under hypoxic condition (1% O_2_ for 4 hours) compared to normoxic condition (21% O_2_). DNA replication was evaluated using an ELISA-based BrdU incorporation assay. (B) The percentage of cybrids invaded in a Matrigel invasion assay. (C) Phase contrast picture of the anchorage independent growth of cybrids with colony formation assay in soft agar. (D) Mean (± SD) number of colonies with each cybrids. (E) Anticancer drug resistance of cybrids: Percentage of viable cybrid cells detected MTT assay after treatment with doxorubicin in normoxic (crossed bar) and hypoxic (doted bar) conditions for 24 hours. (B) Western blot analysis of apoptotic markers cleaved caspase 3 and PARP-1 after treatment with vehicle or doxorubicin (DOX) for 24 hours. β-actin was used as loading control. (* = P<0.05, 10A/143B vs 143B/143B and 468/143B; ^†^ =  P<0.05, 468/143B vs 143B/143B and ^#^ = P<0.05, 10A/143B doxorubicin after normoxic vs hypoxic condition).

In solid tumors, cancer cells have the capacity to proliferate and maintain viability under hypoxic conditions due to the reduced vascularization and decreased blood supply in the tumor tissues. We compared the cell proliferation in cybrids under hypoxic conditions. Interestingly, even under the uniform nuclear background, cybrids with non-cancerous mitochondria showed significantly decreased cell proliferation in hypoxic conditions (Fig. 2A).

### *In vitro* Tumorigenic Properties

The ability to invade cell matrix and to grow under anchorage independent conditions are some of the cancer traits. The nuclear donor 143B osteosarcoma cell line is highly tumorigenic and metastatic in nature. We first analyzed the invasion potential of cybrids using Matrigel invasion chamber. As shown in Fig. 2B, Matrigel invasion assay suggested significantly lower invasion index for 10A/143B cybrids compared to the cybrids with cancer derived mitochondria. We then analyzed the anchorage independent growth potential using *in vitro* colony formation assay in soft agar. Even under uniform metastatic nuclear background, cybrids containing non-cancerous mitochondria formed significantly less colonies compared to cybrids with cancer mitochondria. The number of colonies in cybrids with moderately metastatic MDA-MB-468 cells was in between the 10A/143B and 143B/143B cybrids (Fig. 2C and D).

### Resistance to Anticancer Drug Therapy

Resistance to cell death from anticancer drug is one of the major causes of cancer treatment failure. Most of the conventional chemotherapeutic agents kill via the mitochondrial pathway of apoptosis. Since doxorubicin is often used in the treatment of solid tumors, including breast, liver, and bone tumors, the cell viability after the anticancer drug treatments was analyzed in cybrids under normoxic and hypoxic conditions. Cell viability analysis suggested that compared to 143B/143B and 468/143B cybrids with cancer mitochondria, cybrids containing non-cancerous MCF10A mitochondria had significantly increased cell death after anticancer drug treatment in both normoxic and hypoxic conditions (Fig. 2E). The increased cell death after anticancer therapy was further confirmed by the increased expression of apoptotic markers activated caspase 3 and cleaved PARP in doxorubicin treated 10A/143B cybrids (Fig. 2F). These results suggest that mitochondrial characteristics play significant contribution to the response to anticancer drug therapy.

### *In vivo* Tumor Growth

**Figure 3:**
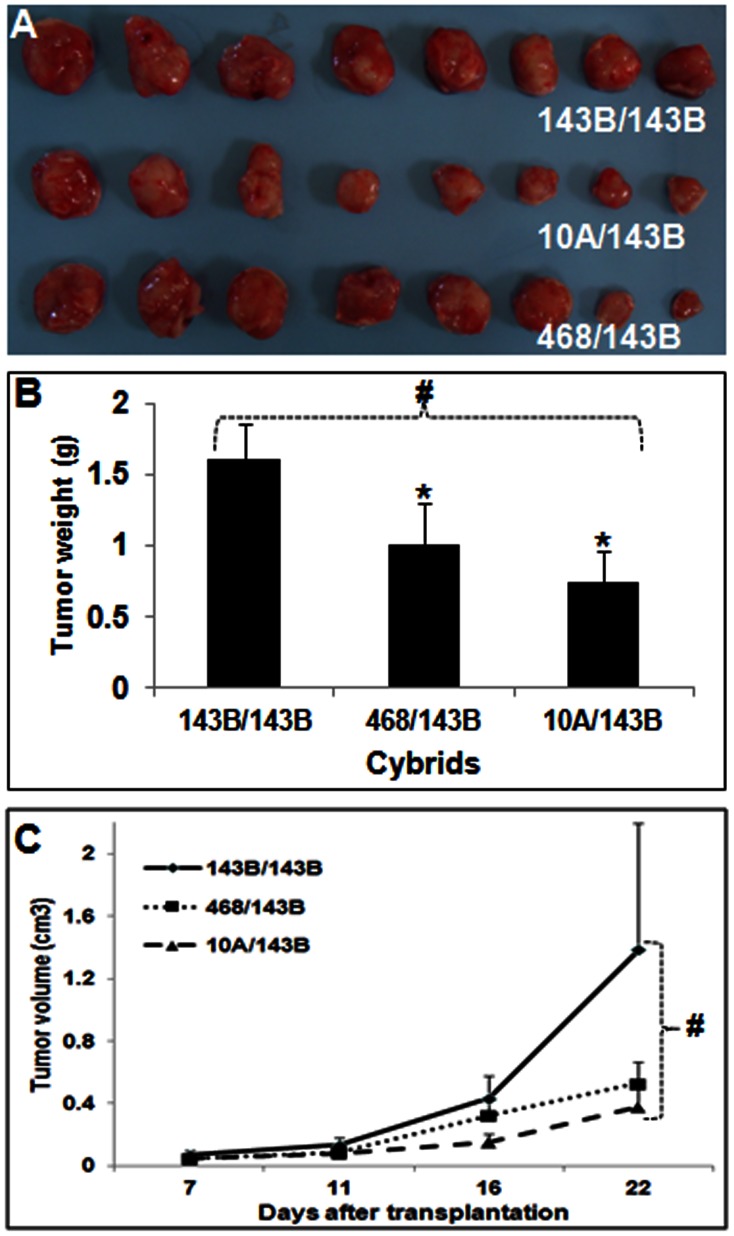
Inhibition of in vivo tumor growth by benign mitochondria. (A) Photograph of the tumors formed with different cybrids after implantation in to nude mice. (B) The mean (SEM) weight of the tumors after their surgical removal. (C) The mean (±SEM) volume of *in vivo* tumor growth at different days after transplanting the cybrids. (* = P<0.05 t-test compared to 143B/143B and # = P<0.01 in ANOVA).

The *in vitro* analyzes strongly suggested the inhibition of oncogenic properties of aggressive 143B cells by the introduction of non-cancerous mitochondria. These findings were further confirmed using an *in vivo* nude mice model. As expected, all cybrids formed tumors under the highly tumorigenic and metastatic 143B nuclear background. However, as observed with *in vitro* colony formation assay, the tumor growth was significantly inhibited in 10A/143B cybrids containing non-cancerous mitochondria compared to 143B/143B cybrids with cancerous mitochondria (Fig. 3A). Tumor weight and size were significantly lower in cybrids with mitochondria derived from benign MCF10A cells (Fig. 3B and C). As observed in the *in vitro* colony formation assay, the tumor size and growth in cybrids with mitochondria derived from moderately cancerous MDA-MB-468 breast cancer cells were in between the 143B/143B cybrids and 10A/143B cybrids. Altogether, our *in vitro* and *in vivo* studies strongly suggest that the mitochondria-nuclear cross talk from non-cancerous mitochondria can inhibit the tumor properties of a highly metastatic cell.

### Mitochondrial Retrograde Signaling from Benign Mitochondria Suppress Several Oncogenic Pathways

**Figure 4:**
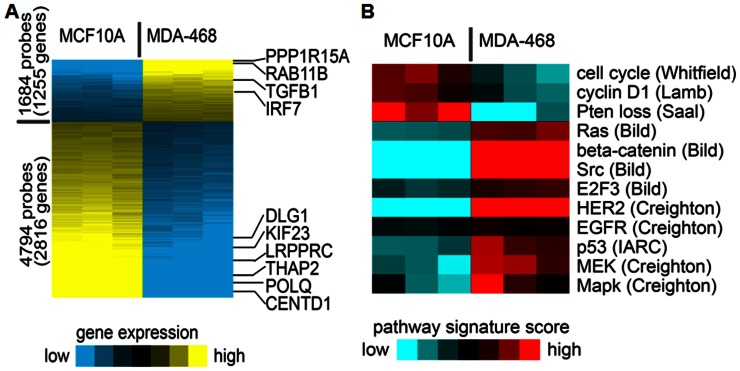
Microarray analysis of cybrids. (A) Comparison of 10A/143B vs 468/143B cybrids with defined genes significant with t test P<0.01, fold change>2 or <0.5. Yellow, higher expression. Genes validated by RT-PCR are indicated. (B) Pathway signature analysis of the expression dataset showing putative differential pathway activation levels (signature names in italics denote differences between the sample groups with P<0.01, t-test). Red, higher inferred pathway signature activity. Pathway signatures were defined using previous studies, indicated in parentheses [24].

Mitochondrial retrograde signaling is a pathway of communication from mitochondria to the nucleus under normal and pathophysiological conditions. To understand the mitochondrial retrograde regulation of nuclear genes by cancerous and non-cancerous mitochondria, we used microarray analysis. Gene expression profiles were compared and bioinformatically analyzed the oncogenic pathways for cybrids with mitochondria from benign breast epithelium (10A/143B) and breast cancer cell (468/143B). Interestingly, even under the same nuclear background, 6478 probe sets representing 4071 unique genes were significantly altered in 10A/143B cybrids compared to 468/143B cybrids (Fig. 4A). Among significantly altered genes, 4794 probe sets representing 2816 unique genes got up-regulated and 1684 probe sets representing 1255 unique genes down regulated in cybrids with MCF10A mitochondria. Further analysis of oncogenic pathways suggest that many oncogenic pathways including RAS, HER2, SRC and P53 pathways are down regulated in MCF10A/143B cybrids with non-cancerous mitochondria (Fig. 4A). Some of the tumor suppressor genes including RB1, PTEN and VHL significantly increased in MCF10A/143B cybrids with benign mitochondria. Microarray data for randomly selected genes were validated by q-RT-PCR analysis (Figure S1). Thus, the microarray analysis strongly suggests that even under a highly aggressive cancer nuclear background, mitochondria-nuclear cross talk from non-cancerous mitochondria can suppress several oncogenic pathways and make the cells less cancerous.

Although for more than 3 decades, tumors are regarded as diseases of mutated oncogenes, alternative “metabolic” models also have been proposed to play a role in the development and progression of cancer. Mitochondrial dysfunction is considered as one of the most common and consistent phenotypes of cancer cells. In recent years, a growing stream of documents is linking cancer genes with energy metabolism [26]. Numerous differences in the molecular composition of the mitochondrial inner membrane between normal and cancer cells have also been reported. Analysis of the inner membrane lipid composition of various tumor mitochondria has indicated elevated levels of cholesterol, varying total phospholipid content, and/or changes in the amount of individual phospholipids relative to normal controls [27]. Our results demonstrated that even though genes that encode most mitochondrial proteins are located in the nucleus, introduction of mitochondria derived from non-cancerous cell to a cancer nuclear environment resulted in suppression of oncogenic pathways and altered cancer properties. Several reports postulated that ROS might be the mediator of the defective mitochondria in cancer [11], [25]. However, factors other than ROS from cancer mitochondria may also play a role in the tumor characteristics. It is also possible that as observed with cellular reprogramming, mitochondria from different cellular environment may have been programmed in certain way and it can carry over some of its effect to the next generation regardless of the nuclear background. Recent studies have suggested that altered mitochondria can affect epigenetic modifications in nuclear genes including DNA methylation [28], [29]. Such epigenetic alterations including DNA and chromatin modifications and signaling through small RNA may contribute to the maintenance of mitochondria mediated oncogenic transformation for generations.

Most of the cybrid studies focused on somatic mutations in mitochondria genome and its functional significance. Many experiments have been conducted to investigate the role of specific mtDNA mutations in cancer [3], [30]–[36]. Although somatic mtDNA changes are common features of cancers and some evidence suggests that certain mtDNA mutations do indeed play a function in the development of cancer, the effect of cancer mitochondria on tumorigenesis remains largely unclear [5], [25]. We did not observe any known pathogenic mutational difference between 143B and MCF10A cells. However, although most of the mitochondrial mutations are individually not deleterious, there is a possibility that combination of different levels of mutations collectively may contribute to the protein stability and/or retrograde signaling.

Resistance to cell death from anticancer drug is one of the major causes of cancer treatment failure. Most of the conventional chemotherapeutic agents kill via the mitochondrial pathway of apoptosis. Mitochondrial pathway of apoptosis behaves as a critical phenomenon, in which there is a rapid, binary transition from a normally functioning cell to the rapid execution of a program of cell death [37]. A recent exciting study suggested that the differences in response to chemotherapeutic treatment might be due to the differences of pretreatment in the readiness of tumor cells to undergo apoptosis, a measurable property that they call “mitochondrial priming” [38]. According to their finding, differential ‘mitochondrial priming’ is an important mechanism underlying the therapeutic index of conventional chemotherapy [38]. Anti-cancer agents specifically targeting cancer cell mitochondria are named as ‘mitocans’ [39]–[42]. These structurally distinct molecules that share one unifying feature: they all exert anti-cancer properties based on their ability to induce apoptosis in malignant cells by targeting mitochondria. For example, vitamin E analogues are potent novel anticancer drugs that target mitochondria and act through its pro-apoptotic and anti-cancer activity [41]. Current developments in mitochondria research may provide novel mitocans or allow repositioning some of the already available mitochondria targets for cancer therapy alone or in combination with other anticancer drugs.

To conclude, the present study clearly suggests that oncogenic properties of an aggressive cancer cell can at least partially reversed by the cross-talk from a non-cancerous mitochondria by suppressing several oncogenic pathways. The findings and pathways identified in this study open up new avenues to identify novel drug targets as well as to supplement currently available chemotherapeutic drugs with mitochondria targeting agents. Further studies are necessary using the pathways proposed from the microarray analysis and cybrids with non-cancerous or moderate breast cancer nuclear backgrounds.

## Supporting Information

Figure S1 **Confirmation of microarray data.** qPCR confirmation of microarray data in randomly selected up and down regulated genes in MCF10A/143B cybrids compared to 468/143B cybrids.
(TIF)

Table S1 **Primers used for qRT-PCR confirmation of microarray data.**
(PDF)
