# Correction: Crosstalk from Non-Cancerous Mitochondria Can Inhibit Tumor Properties of Metastatic Cells by Suppressing Oncogenic Pathways

**DOI:** 10.1371/journal.pone.0221671

**Published:** 2019-08-22

**Authors:** Benny Abraham Kaipparettu, Yewei Ma, Jun Hyoung Park, Tin-Lap Lee, Yiqun Zhang, Patricia Yotnda, Chad J. Creighton, Wai-Yee Chan, Lee-Jun C. Wong

There are errors in Fig 1E. The panels for Core 2 (complex III) and for F1α (complex V) are incorrect. A duplicate of the Ip (complex II) panel was used in error for Core 2 (complex III). Bands showing Core 2 (complex III) were used in error for F1α (complex V).

The authors apologize for these errors. A corrected Fig 1 in which replacement band images are taken from the original experiment is provided here.

**Fig 1:**
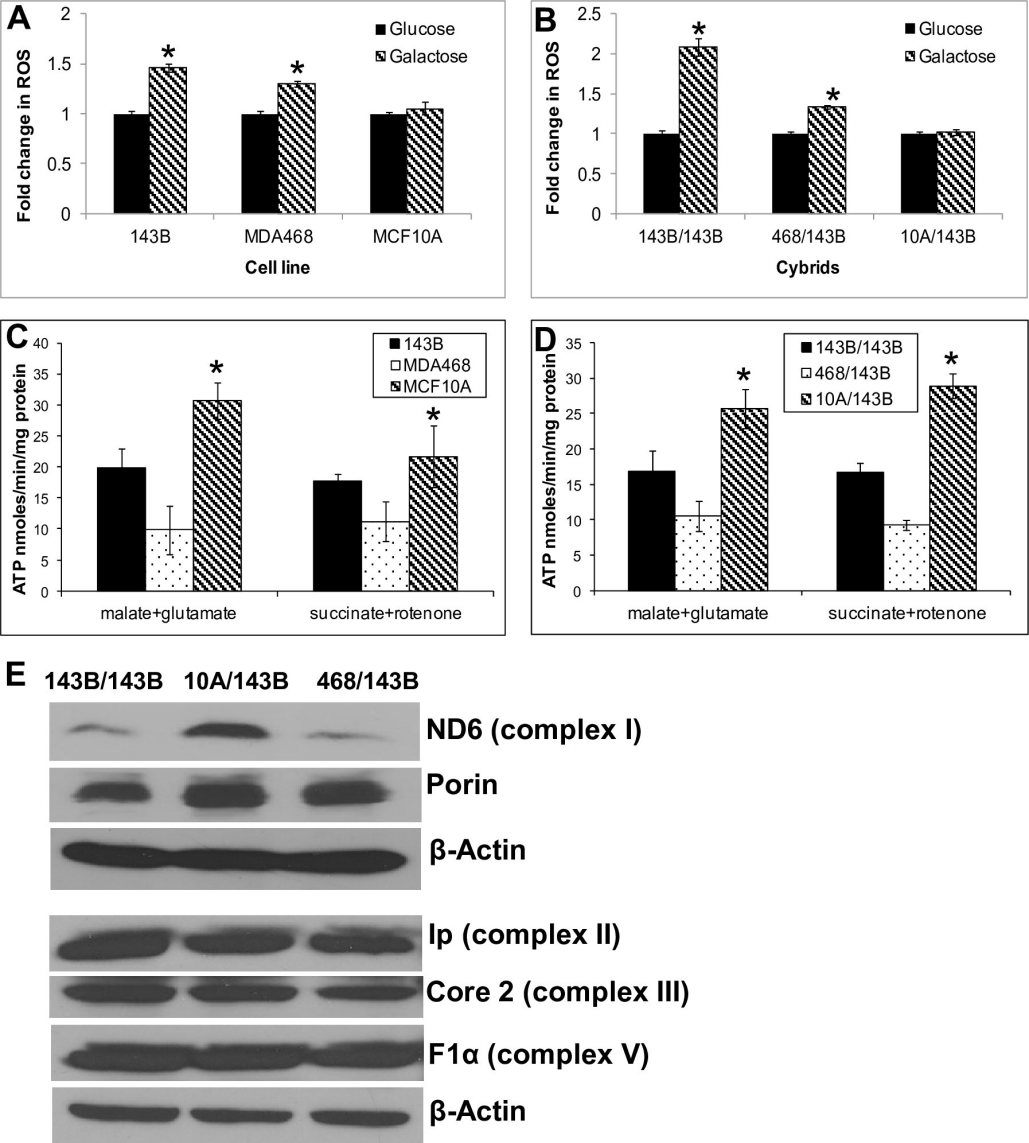
ROS production and ATP synthesis. (A) ROS production in parental cells. After Cells were grown in DMEM-glucose or DMEM-galactose medium for 24 h and ROS were measured using DCFH-DA fluorescent dye. Data expressed as DCF fluorescence/mg protein. (B) ROS production in cybrid cells. (C) Mitochondrial ATP synthesis rate in parental cells. ATP synthesis was driven by complex I substrates (glutamate plus malate) or complex II substrate (succinate) in the presence of complex I inhibitor (rotenone). The rates of ATP synthesis are expressed as nmoles ATP/min/mg protein. (D) Mitochondrial ATP synthesis rate in cybrids. (E) Western blot analysis of representative protein subunits of the mitochondria respiratory complexes in cybrids. Core 2 (complex III) band is taken from the same blot as Ip (complex II), F1α (complex V) and the corresponding β-actin loading control, but after a higher exposure. The ND6 (complex I) bands are taken from the same blot as the corresponding β-actin loading control. β-actin and porin were used as loading controls for the nuclear and mitochondrial proteins respectively.

The underlying blot images for Fig 1E are provided below as Supporting Information, along with the underlying data for the charts in Fig 1 as summary mean and SD.

For Fig 1E, complexes II, III, and V were analyzed in a single gel, along with a β-actin loading control, which was analyzed in the same blot after stripping and re-blotting. The Core 2 (complex III) panel used a higher exposure than the other panels.

ND6 and β-actin were analyzed in the same blot, along with porin which was analyzed in the same blot after stripping and re-blotting.

Fig 4 reports the results of a microarray experiment. The raw data for the microarray experiment are deposited at GEO, accession GSE128610. The underlying data for other parts of the article are not available at the time of publication of this notice.

## Supporting information

S1 File Fig 1 Chart Data. (PDF)

S2 File Underlying Blots for Fig 1E. (PDF)
