# Supplementary figures and images for: Crosstalk from Non-Cancerous Mitochondria Can Inhibit Tumor Properties of Metastatic Cells by Suppressing Oncogenic Pathways
Source: PLoS One. 2013 May 9;8(5):e61747. doi: 10.1371/journal.pone.0061747 (PMC3650012; doi:10.1371/journal.pone.0061747)

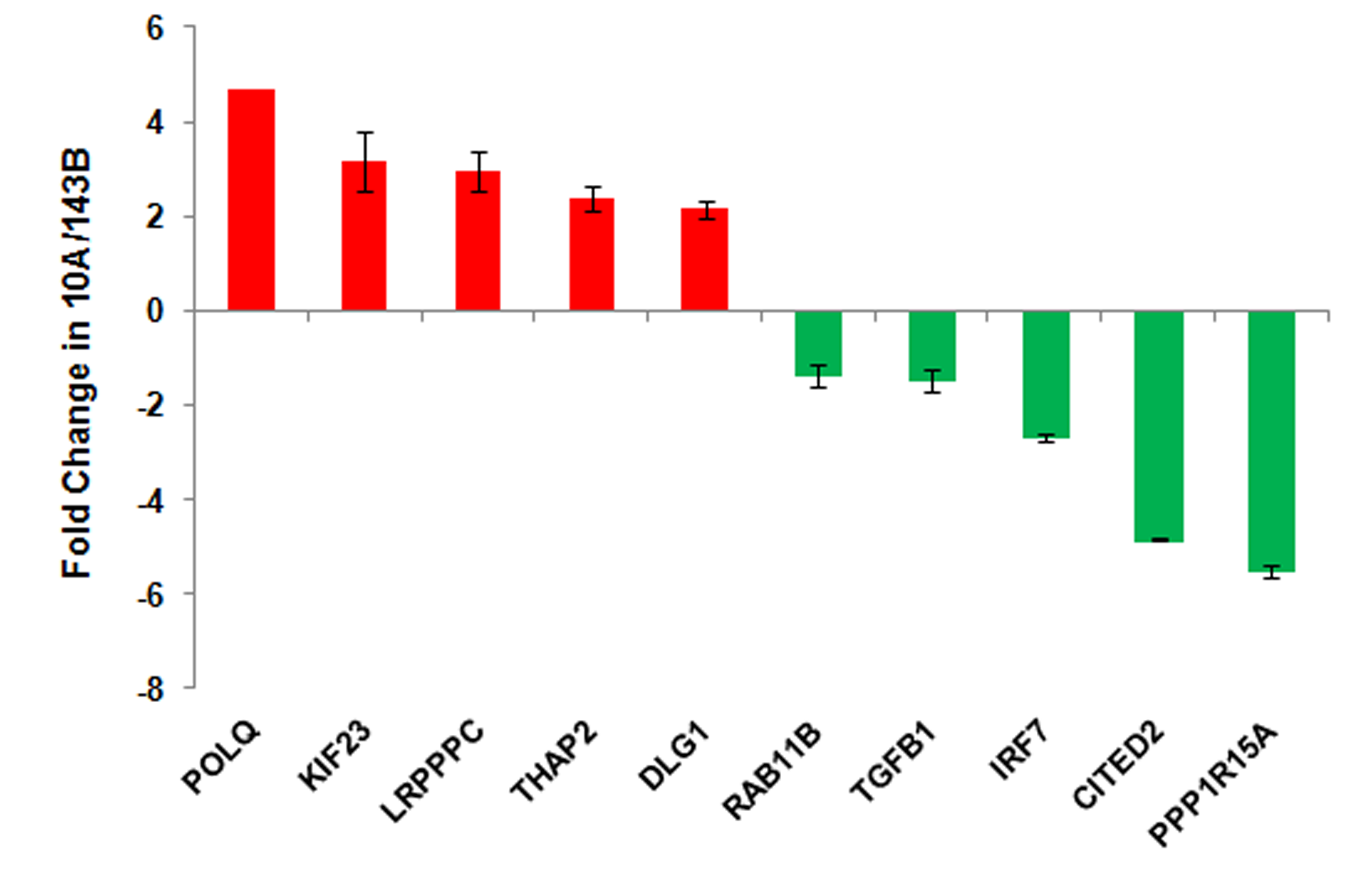

Supplement: Figure S1 — Confirmation of microarray data. qPCR confirmation of microarray data in randomly selected up and down regulated genes in MCF10A/143B cybrids compared to 468/143B cybrids. (TIF) [file pone.0061747.s001.tif]
