# Supplementary material for: Crosstalk from Non-Cancerous Mitochondria Can Inhibit Tumor Properties of Metastatic Cells by Suppressing Oncogenic Pathways
Source: PLoS One. 2013 May 9;8(5):e61747. doi: 10.1371/journal.pone.0061747 (PMC3650012; doi:10.1371/journal.pone.0061747)
Supplement: Table S1 — Primers used for qRT-PCR confirmation of microarray data. (PDF) [file pone.0061747.s002.pdf]

Supplementary Table-1: qRT-PCR primers used for microarray validation

**Gene**	**Forward Primer**	**Reverse Primer**
PPP1R15A	GCCCAGAAACCCCTACTCAT	GCCAGGAAATGGACAGTGAC
RAB11B	GGGACGACGAGTACGACTACC	CTCCAGGTTGAACTCGTTGC
TGFB1	CCAAAGGAAAATCTGTGGCA	TTGAGAGTGGTAGGGCTGCT
IRF7	GTGATGCTGCGGGATAACTC	TCAGTCTGGTCCGTGCCT
DLG1	TTCAGAGACACTGCCAAGCA	TCATTTGTGATTTGTGGGGA
KIF23	GAACTAACCGGACCAGAGCA	TGGTTCTCTCTTAGGACATCCAT
LRPPRC	CATGCCGCCTCCTATCTG	TTTTCTTTGGCAGCAATGG
THAP2	GGGCTGTGCCACTACCTACA	AAAATTTTTGCGCCTAACCA
POLQ	TTTTGCTGACCTGCAAAGAG	TCCAATCCTCAAACATAGGTG
CENTD1	TGAACCTGGCTCTGCTTACC	TTCCTTCTTTGATGCTTCCC
B2M	TAGCTGTGCTCGCGCTACT	TCTCTGCTGGATGACGTGAG
