# Supplementary material for: Correction: Crosstalk from Non-Cancerous Mitochondria Can Inhibit Tumor Properties of Metastatic Cells by Suppressing Oncogenic Pathways
Source: PLoS One. 2019 Aug 22;14(8):e0221671. doi: 10.1371/journal.pone.0221671 (PMC6706049; doi:10.1371/journal.pone.0221671)
Supplement: S1 File — (PDF) [file pone.0221671.s001.pdf]

Figure-1 A-D data points

A

	Fold		SD	
	Glucose	Galactose	Glucose	Galactose
143B	1	1.46359	0.02667	0.04383
MDA468	1	1.30389	0.02591	0.02965
MCF10A	1	1.05464	0.01886	0.06461

B

	Fold		SD	
	Glucose	Galactose	Glucose	Galacto se
143B/143B	1	2.09088	0.04553	0.11085
468/143B	1	1.33524	0.02703	0.01895
10A/143B	1	1.02099	0.022	0.02981

C

	ATP		SD	
	M + G	S + R	M + G	S + R
143B	19.754	17.675	3.16448	1.24242
MDA468	9.81861	11.2125	3.868	3.1585
MCF10A	30.684	21.6573	2.85744	4.96179

D

	ATP		SD	
	M + G	S + R	M + G	S + R
143B/143B	16.8188	16.6817	2.97389	1.30346
468/143B	10.5006	9.26333	2.1309	0.64421
10A/143B	25.7505	28.9258	2.7681	1.74168

M + G: Malate + Glutamate   S + R: Succinate + Rotenone
