# Supplementary material for: Correction: Crosstalk from Non-Cancerous Mitochondria Can Inhibit Tumor Properties of Metastatic Cells by Suppressing Oncogenic Pathways
Source: PLoS One. 2019 Aug 22;14(8):e0221671. doi: 10.1371/journal.pone.0221671 (PMC6706049; doi:10.1371/journal.pone.0221671)

Original blots for ND6 and β-Actin with different exposures

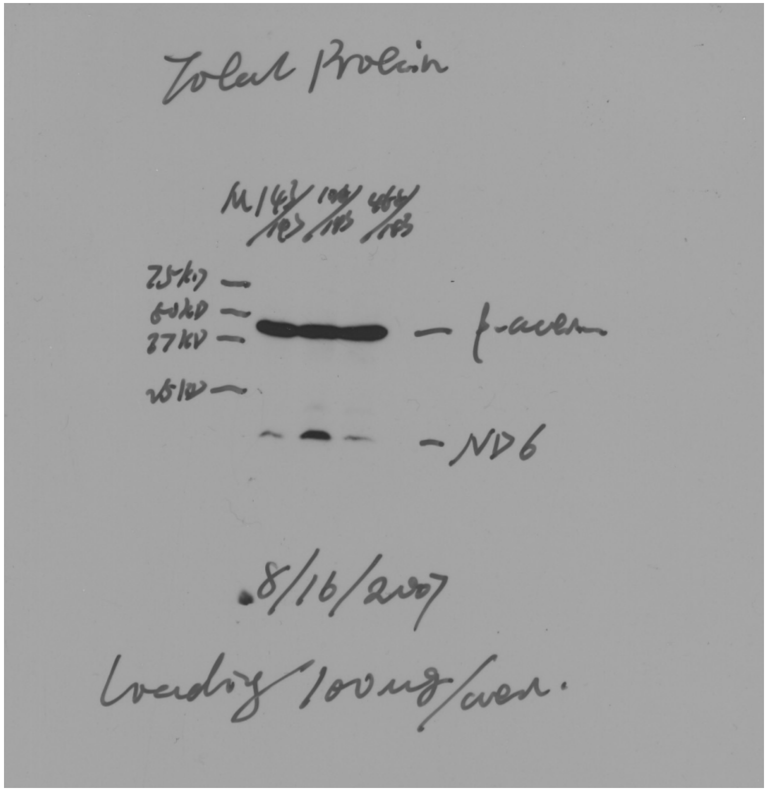

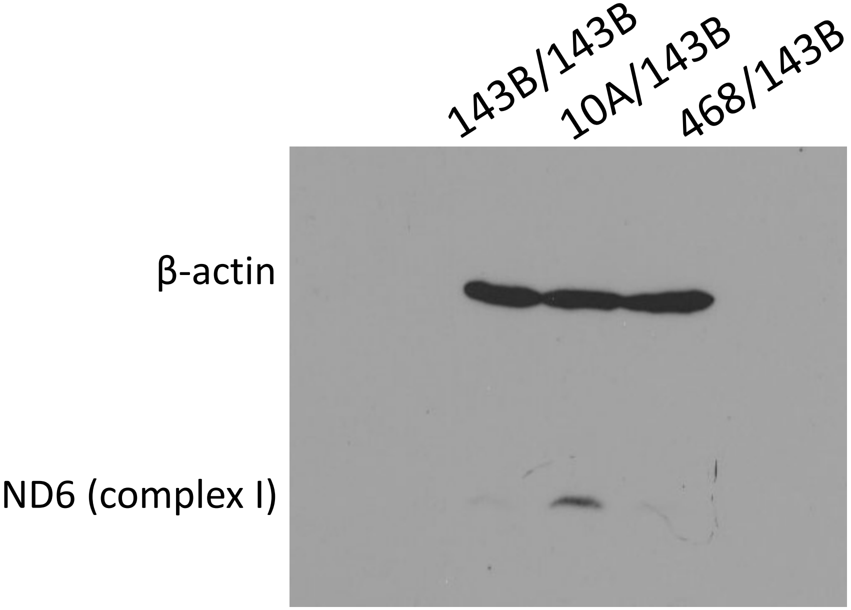

Original blots for Porin

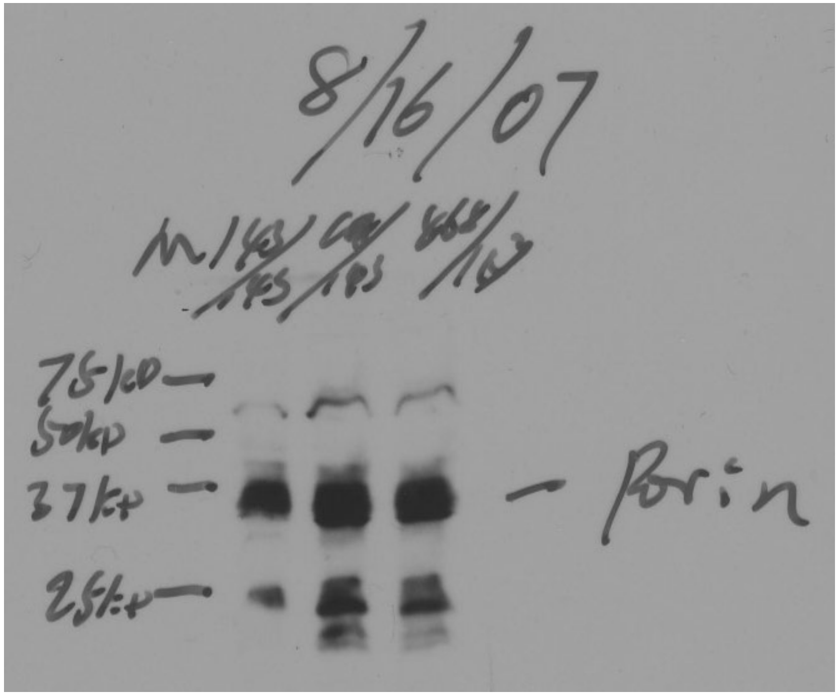

Original blots for Complexes with different exposures

A

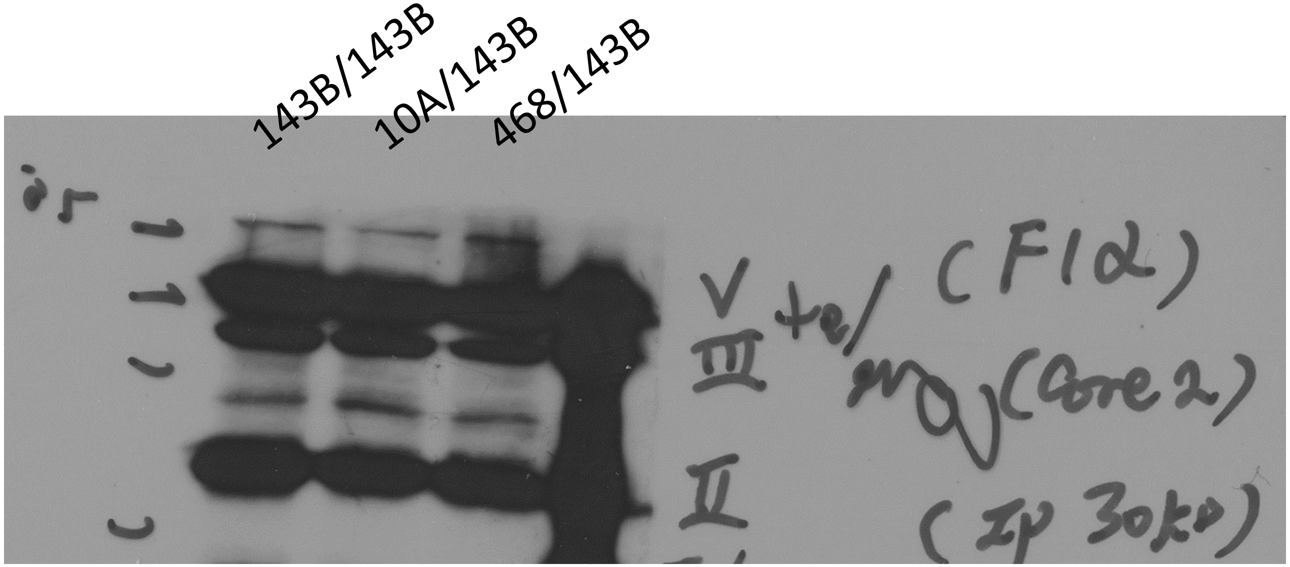

B

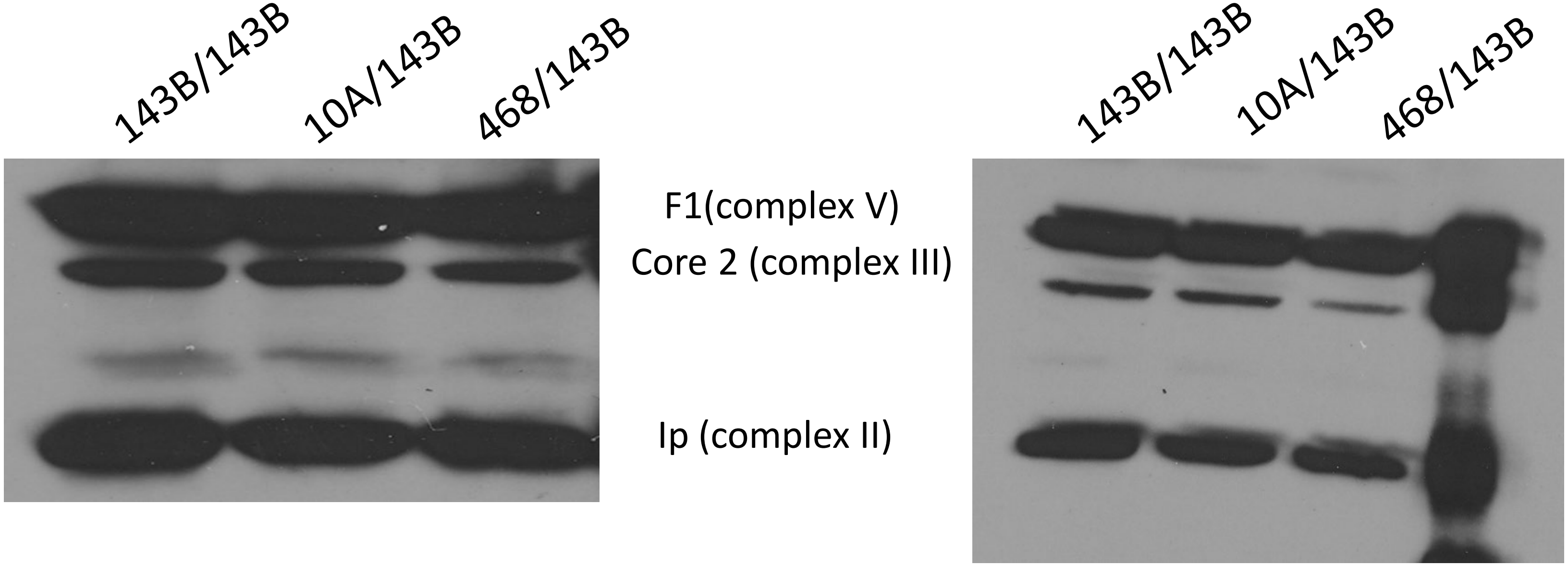

Original blots for β-Actin for the complexes

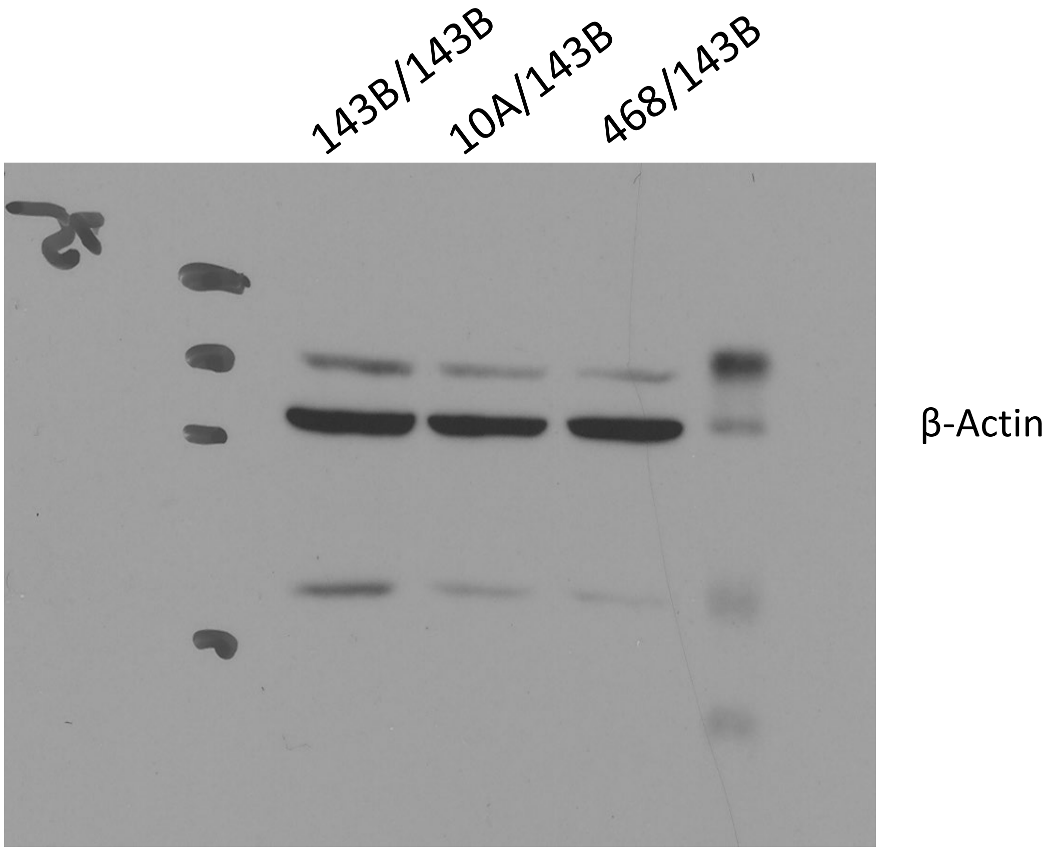

Supplement: S2 File — (PDF) [file pone.0221671.s002.pdf]
